# Global Prevalence of Nosocomial Multidrug-Resistant *Klebsiella pneumoniae*: A Systematic Review and Meta-Analysis

**DOI:** 10.3390/antibiotics10121508

**Published:** 2021-12-08

**Authors:** Nur Ain Mohd Asri, Suhana Ahmad, Rohimah Mohamud, Nurmardhiah Mohd Hanafi, Nur Fatihah Mohd Zaidi, Ahmad Adebayo Irekeola, Rafidah Hanim Shueb, Leow Chiuan Yee, Norhayati Mohd Noor, Fatin Hamimi Mustafa, Chan Yean Yean, Nik Yusnoraini Yusof

**Affiliations:** 1 Health Campus, Institute for Research in Molecular Medicine (INFORMM), Universiti Sains Malaysia, Kubang Kerian 16150, Malaysia; ain.asri@live.iium.edu.my (N.A.M.A.); fatihah@usm.my (N.F.M.Z.); fatinmustafa@usm.my (F.H.M.); 2 Department of Plant Sciences, Kuliyyah of Science, International Islamic University Malaysia, Kuantan 25200, Malaysia; nurmardhiah.hanafi@live.iium.edu.my; 3 Department of Immunology, School of Medical Sciences, Universiti Sains Malaysia, Kubang Kerian 16150, Malaysia; suhanaahmad@usm.my (S.A.); rohimahm@usm.my (R.M.); 4 Department of Medical Microbiology and Parasitology, School of Medical Sciences, Universiti Sains Malaysia, Kubang Kerian 16150, Malaysia; irekeola@student.usm.my (A.A.I.); hanimkk@usm.my (R.H.S.); 5 School of Pharmaceutical Sciences, Universiti Sains Malaysia, Glugor 11800, Malaysia; yee.leow@usm.my; 6 Department of Family Medicine, School of Medical Sciences, Universiti Sains Malaysia, Kubang Kerian 16150, Malaysia; hayatikk@usm.my

**Keywords:** worldwide, prevalence, nosocomial, multidrug-resistant, *Klebsiella pneumoniae*, antibiotic resistance, systematic review, meta-analysis

## Abstract

The emergence of nosocomial multidrug-resistant *Klebsiella pneumoniae* is an escalating public health threat worldwide. The prevalence of nosocomial infections due to *K. pneumoniae* was recorded up to 10%. In this systematic review and meta-analysis, which were conducted according to the guidelines of Preferred Reporting Items for Systematic Review and Meta-Analysis, 1092 articles were screened from four databases of which 47 studies fulfilled the selected criteria. By performing a random-effect model, the pooled prevalence of nosocomial multidrug-resistant *K. pneumoniae* was estimated at 32.8% (95% CI, 23.6–43.6), with high heterogeneity (I^2^ 98.29%, *p*-value < 0.001). The estimated prevalence of this pathogen and a few related studies were discussed, raising awareness of the spread of multidrug-resistant *K. pneumoniae* in the healthcare setting. The emergence of nosocomial multidrug-resistant *K. pneumoniae* is expected to increase globally in the future, and the best treatments for treating and preventing this pathogen should be acknowledged by healthcare staff.

## 1. Introduction

The rise of antibiotic resistance among infectious disease-causing bacteria, such as *Klebsiella pneumoniae* (*K. pneumoniae*), is an escalating public health threat around the world. It does not only increase the morbidity and mortality rates in patients, but also prolongs hospital stays and increases the treatment costs [1]. *K. pneumoniae* is a gram-negative, encapsulated, rod-shaped, non-motile bacterium [2], and an important opportunistic pathogen from the Enterobacteriaceae family that causes a large number of nosocomial infections, particularly in developing countries [3]. As stated by Ghashghaee et al. [4], the global prevalence of hospitalized patients exposed to nosocomial infections was 8.7% and the effects could be more burdensome for patients suffering from cancer, organ transplants, and surgery. Additionally, up to 10% of the nosocomial infections are caused by *K. pneumoniae* [5].

*K*. *pneumoniae* can be found almost anywhere in the body and the most frequent infections that occur in humans are urinary tract infections (UTIs), meningitis, respiratory tract infections (RTIs), pneumonia, bloodstream infections (BSIs), and surgical site infections (SSIs). Neonates, the elderly, and immunocompromised patients are the most vulnerable groups to *K*. *pneumoniae* infection [6]. Neonates are at risk for *K*. *pneumoniae* infections due to the undeveloped body physiology [7]. As recorded, 1.6 million neonatal death occurred every year due to sepsis, predominantly in middle and low income countries [8]. Immunocompromised patients who are hospitalized and suffer from underlying chronic illnesses as well as the elderly are prone to be infected with *K. pneumoniae* as their immune system’s defences are low [9].

Antibiotics have previously been acknowledged to have saved a large amount of lives, but the emergence of resistance towards antibiotics has threatened healthcare systems worldwide. Antibiotic resistance can be classified into three groups: Multidrug-resistant (MDR), extensively drug-resistant (XDR), and pandrug-resistant (PDR). These three types of antibiotic resistance are classified based on their susceptibility to several classes of antibiotics. MDR *K*. *pneumoniae* developed resistance to at least one agent from three or more antibiotic classes. Meanwhile, XDR *K*. *pneumoniae* strains are resistant to at least one agent in all of the antibiotic classes except two or fewer classes. Lastly, *K*. *pneumoniae* which is resistant to all of the agents in all antibiotic classes is classified as PDR, and this type of antibiotic resistance is the worst infection scenario in the healthcare setting [10]. The emergence of MDR *K*. *pneumoniae* arose due to the extensive use of antibiotics when treating hospitalized patients [6], making it a challenge to treat and prevent the spread of the infection in the hospital setting.

Extended spectrum beta lactamase-producing *K. pneumoniae* (ESBL-KP) and carbapenem-resistant *K. pneumoniae* (CRKP) strains have been reported to cause severe infections in humans. Most of the ESBL-KP and CRKP have contributed to the emergence of multidrug-resistance strains, diminishing treatment options for patients [6,11]. Furthermore, both resistance (*bla_TEM_*, *bla_SHV_*, *bla_KPC_*, *bla_OXA_*, *qnrB1*, *oqxA*, *dfrA12*, *sul2*, *fosA,* and *mgrB*) [12], and virulence genes *(iro*, *kpn*, *fimH, mrkD*, *entB*, *traT*, *rmpA*, *fyuA*, *magA,* and *hlyA*) [13] encoded in the bacterium genome played significant factors in the emergence of MDR *K*. *pneumoniae.*

Nosocomial infections often arise during the process of receiving a health treatment when an infection is absent upon hospital admission, but it can appear up to 48 h after admission [14]. The infections may happen in various settings of healthcare delivery, including hospitals, long-term care facilities, and ambulatory settings. In addition, it may show up following a discharge from the hospital. Nosocomial infections not only affect patients, but can also affect the healthcare staff [15].

The aim of this systematic review and meta-analysis is to estimate the prevalence of nosocomial infections due to multidrug-resistant *K*. *pneumoniae* worldwide and portray the reality of the emergence of these fatal strains.

## 2. Results

### 2.1. Search and Screening Results

**Figure 1:**
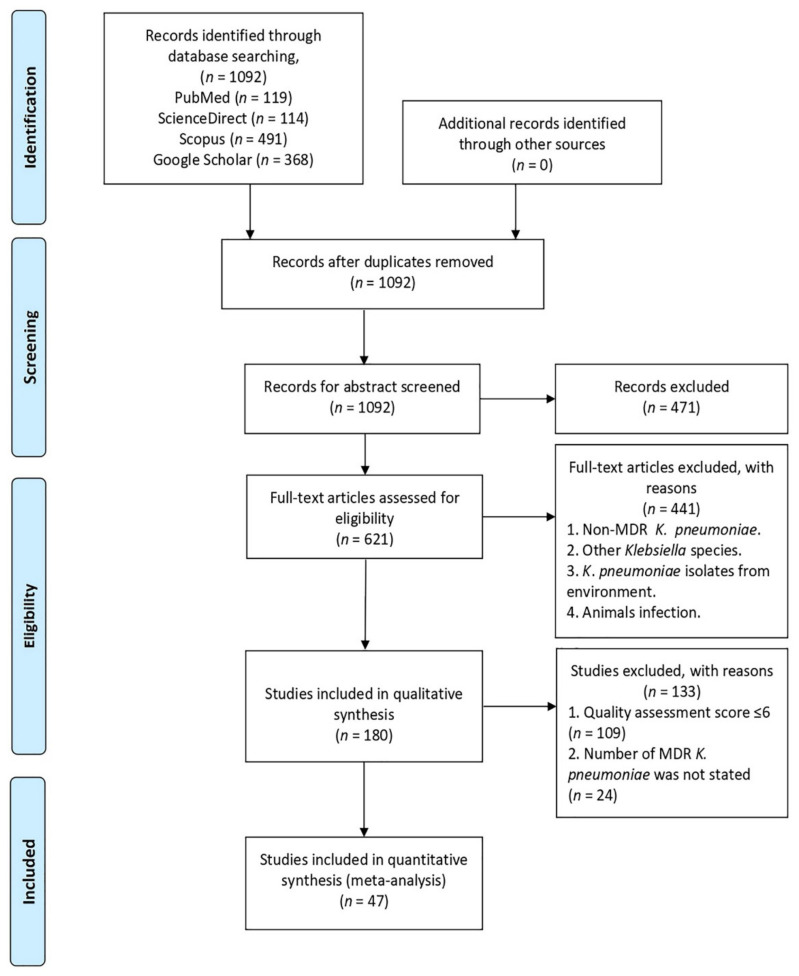
PRISMA flow diagram illustrating the selection process of the studies in this analysis.

A total of 1092 studies were identified from four electronic databases (PubMed, ScienceDirect, Google Scholar, Scopus). The screening of abstracts was done on 1092 articles based on the inclusion and exclusion criteria. As a result, 621 articles were included in full text-screening. Then, 441 articles were excluded after full-article assessment based on the same inclusion and exclusion criteria. In total, 180 articles were selected for data extraction prior to removing 109 articles from this review, due to the high and moderate risk bias based on the quality assessment score (≤6 score) (Appendix A; Table A1). Thereafter, from the 71 articles selected, only 47 studies portrayed all of the selected criteria, and they were included in this analysis (Figure 1).

### 2.2. Characteristics of the Included Studies

**Figure 2:**
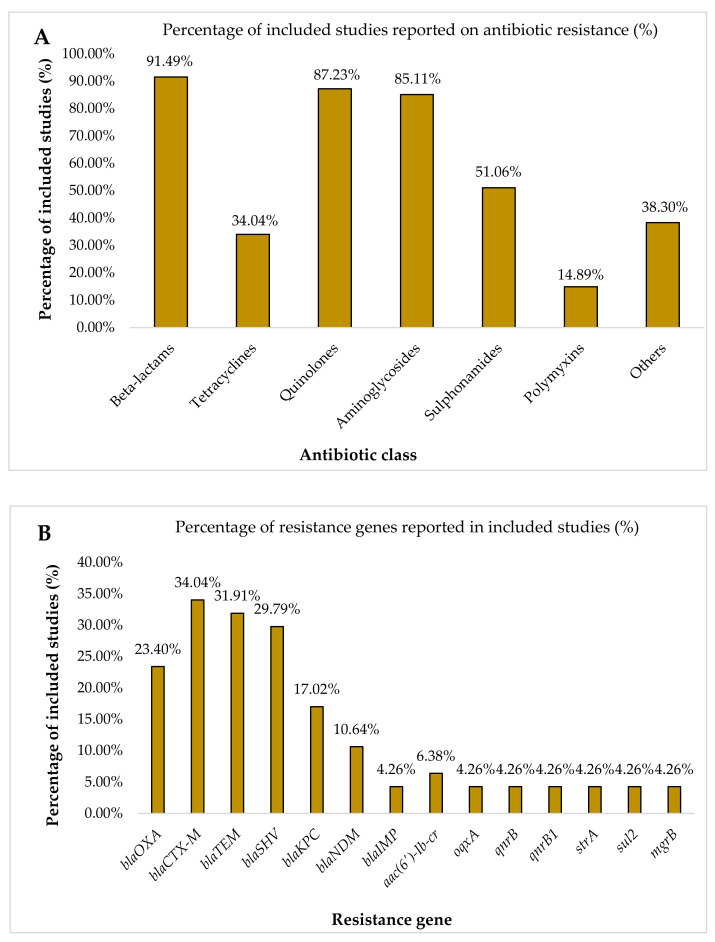
Percentage of resistance according to (**A**) antibiotic classes and (**B**) resistance genes in the included studies.

The recorded antibiotic resistance profiles from the included studies varied between one another. Based on the 47 studies, 43 (91.49%), 41 (87.23%), 40 (85.11%), 24 (51.06%), 18 (38.30%), 16 (34.04%), and 7 (14.89%) of the included studies showed consistent resistance to beta-lactams, quinolones, aminoglycosides, sulphonamides, other classes of antibiotics, tetracyclines, and polymyxins, respectively (Figure 2A). Beta-lactams can be divided into six classes which are penicillins, penicillins/beta-lactamase inhibitor, cephalosporins, cephalosporins/beta-lactamase inhibitor, carbapenems, and monobactams (Appendix A; Table A2). In these studies, the examples of the other classes of antibiotics were chloramphenicol, trimethoprim, nitrofurantoin, fosfomycin, and rifampin, while four (8.51%) studies did not record the antibiotic resistance profile as they only stated the number of nosocomial MDR *K*. *pneumoniae* cases. Information on the characteristics of the included studies is presented in Table 1. Genes encoded for antibiotic resistance were reported in 24 (51.06%) of the included studies. The majority of the reported antibiotic resistance genes were genes encoded for β-lactamases enzymes, which account from 22 studies (46.81%). According to Figure 2B, the percentages of the included studies that recorded the resistance genes for the class of beta-lactams were *bla_OXA_* (23.40%), *bla_CTX-M_* (34.04%), *bla_TEM_* (31.91%), *bla_SHV_* (29.79%), *bla_KPC_* (17.02%), *bla_NDM_* (10.64%), and *bla_IMP_* (4.26%), while these three genes, *bla_DHA_*, *bla_VIM_*, and *bla_CMY_*, were recorded once in three different studies (2.13%). Furthermore, the other resistance genes were also recorded in the included studies, such as *aac(6′)-Ib-cr* (6.38%), and each of these resistance genes, *oqxA*, *qnrB*, *qnrB1*, *strA*, *sul2*, and *mgrB*, have the same percentage which was 4.26%.

### 2.3. Prevalence of Nosocomial MDR K. pneumoniae

**Figure 3:**
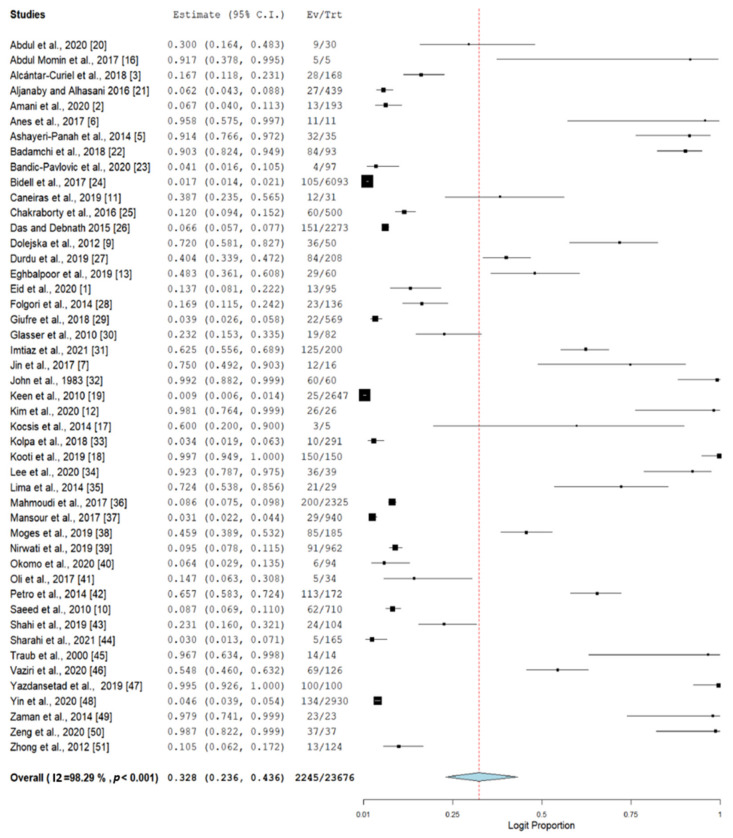
Forest plot of the prevalence of nosocomial MDR *K. pneumoniae*. The prevalence of reported nosocomial MDR *K. pneumoniae* cases was estimated by a random-effect model using the DerSimonian-Laird method of meta-analysis.

The 47 studies included in this meta-analysis comprised a total of 23,676 isolates from nosocomial infections. From these studies, 5822 isolates were positively identified as an infection due to *K. pneumoniae*. Using a random-effect model, the pooled prevalence of nosocomial MDR *K. pneumoniae* was estimated at 32.8% (95% CI, 23.6–43.6), but with high heterogeneity (I^2^ 98.29%, *p*-value < 0.001) (Figure 3).

To investigate the potential sources of heterogeneity, a subgroup analysis from different countries and regions was performed (Table 2). Data recorded from the included articles showed that the studies from Iran (*n* = 10) accounted for the majority of the studies. The highest estimate of 98.1% (95% CI, 76.4–99.9) was observed from South Korea (*n* = 1) and the lowest pooled prevalence was estimated at 3.1% (95% CI, 2.2–4.4) from Tunisia (*n* = 1). Most of the estimates from different countries observed the same heterogeneity as the pooled prevalence (I^2^ 94.38–98.14%). According to regions, studies from the North America region (*n* =5) observed the lowest estimate at 12.9% (95% CI, 3.1–40.3) and studies from the South America region (*n* = 1) gave the highest estimate at 72.4% (95% CI, 53.8–85.6). The studies from the North America region showed a significant estimated prevalence for nosocomial MDR *K. pneumoniae*. 

Another approach that provides a partial explanation of heterogeneity is the analysis of sensitivity and publication bias. In this meta-analysis, sensitivity is assessed by evaluating the impact of small sample size and leave-one-out analysis. Two studies [16,17] with a sample size of 10 and below were excluded and the re-estimated prevalence was 31.5% (95% CI, 22.4–42.2), indicating a slight decline from the original prevalence of 32.8%. Furthermore, the leave-one-out analysis was performed by removing one study at a time, using a random-effects model. The lowest estimate of 30.9%, which was observed following the analysis, was obtained when the study from Kooti et al. [18] was removed. Meanwhile, the highest prevalence of 34.7% was observed when the study from Keen et al. [19] was removed. Overall, the prevalence estimate of nosocomial MDR *K. pneumoniae* was stable.

**Figure 4:**
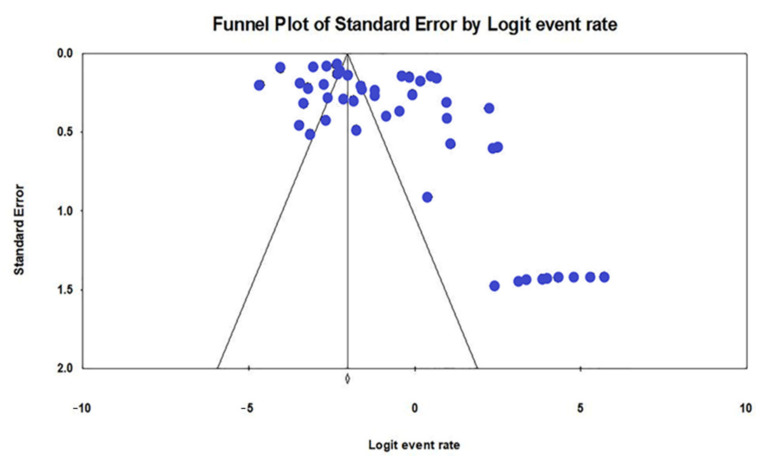
Funnel plot showing the evidence of publication bias with Egger’s test (*p*-value < 0.001).

The selected studies in this review had a good methodological quality according to the JBI Quality Assessment Tool for Prevalence Studies (Appendix A; Table A1). A visual observation of the funnel plot, which was generated for all of the included studies, showed a relatively asymmetrical plot with evidence of publication bias (Figure 4). Furthermore, Egger’s test for the asymmetrical funnel plot revealed a significant publication bias (*p*-value < 0.001).

**Table 1:** Characteristics of the 47 included studies in this analysis.

Author ID	Country	Number of Isolates	Number of *K. pneumoniae*	Number of MDR *K. pneumoniae*	Resistance Profile to Antibiotic Class	Genes Encoded for Antibiotic Resistance
Abdul et al. 2020 [20]	Iraq	30	14	9	Beta-lactams, Quinolones, Aminoglycosides, Sulphonamides.	NR
Abdul Momin et al. 2017 [16]	Brunei	5	5	5	Beta-lactams, Quinolones, Aminoglycosides, Sulphonamides.	*bla_OXA-232_*, *bla_CTX-M-15_*, *bla_TEM-1b_*, *bla_SHV-11_*
Alcántar-Curiel et al. 2018 [3]	Mexico	168	168	28	Beta-lactams, Tetracyclines, Quinolones, Aminoglycosides.	NR
Aljanaby and Alhasani 2016 [21]	Iraq	439	32	27	Beta-lactams, Tetracyclines, Quinolones, Aminoglycosides, Chloramphenicol, Nitrofurantoin.	NR
Amani et al. 2020 [2]	Iran	193	36	13	Beta-lactams, Aminoglycosides, Chloramphenicol, Nitrofurantoin.	*o* *qxA*
Anes et al. 2017 [6]	United Kingdom	11	11	11	Beta-lactams, Tetracyclines, Quinolones, Aminoglycosides, Sulphonamides, Chloramphenicol.	*bla_CTX__-__M__-__15_*, *bla_SHV-12_*, *bla_TEM-1B_*, *oqxAB*, *qnrB*
Ashayeri-Panah et al. 2014 [5]	Iran	35	35	32	Beta-lactams, Quinolones, Aminoglycosides, Sulphonamides, Polymyxin, Nitrofurantoin.	*bla_SHV_*
Badamchi et al. 2018 [22]	Iran	93	93	84	Beta-lactams, Quinolones, Aminoglycosides, Sulphonamides, Rifampin.	*bla_KPC_*
Bandic-Pavlovic et al. 2020 [23]	Croatia	97	8	4	Beta-lactams, Quinolones, Aminoglycosides.	*bla_CTX-M-15_*, *bla_OXA-48_*
Bidell et al. 2017 [24]	United States of America	6093	1039	105	Beta-lactams, Quinolones.	NR
Caneiras et al. 2019 [11]	Portugal	31	31	12	Beta-lactams, Tetracyclines, Quinolones, Aminoglycosides, Fosfomycin.	*bla_TEM-10,_ bla_TEM-24_*, *bla_CTX-M__-__15_*, *bla_KPC-3_*, *bla_SHV-11_*
Chakraborty et al. 2016 [25]	Bangladesh	500	108	60	Beta-lactams, Tetracyclines, Quinolones, Aminoglycosides, Sulphonamides.	NR
Das and Debnath 2015 [26]	India	2273	671	151	NR	NR
Dolejska et al. 2012 [9]	Czech Republic	50	36	36	Beta-lactams, Tetracyclines, Quinolones, Aminoglycosides, Sulphonamides, Chloramphenicol.	*bla_CTX-M-15_*, *bla_TEM-1_*, *bla_OXA-1_*, *aac(6’)-Ib-cr*, *qnrB1*, *strA*, *sul2*, *tet(A)*, *aac(3’)-II*
Durdu et al. 2019 [27]	Turkey	208	208	84	Beta-lactams, Tetracyclines, Quinolones, Aminoglycosides, Sulphonamides, Polymyxin.	NR
Eghbalpoor et al. 2019 [13]	Iran	60	60	29	Beta-lactams, Quinolones, Aminoglycosides, Sulphonamides.	*bla_CTX-M_*, *bla_TEM_*, *bla_SHV_*
Eid et al. 2020 [1]	Egypt	95	22	13	Beta-lactams, Tetracyclines, Quinolones, Aminoglycosides, Chloramphenicol.	NR
Folgori et al. 2014 [28]	Italy	136	37	23	NR	*bla_KPC,_ bla_OXA-48_*
Giufre et al. 2018 [29]	Italy	569	52	22	Beta-lactams, Quinolones, Sulphonamides.	*bla_CTX-M-14_*, *bla_CTX-M-15_*, *bla_TEM-24_*, *bla_TEM-52_*, *bla_SHV-12_*, *bla_KPC-3_*
Glasser et al. 2010 [30]	United States of America	82	22	19	Beta-lactams, Quinolones, Aminoglycosides.	NR
Imtiaz et al. 2021 [31]	Pakistan	200	200	125	Beta-lactams, Quinolones, Aminoglycosides, Polymyxin.	*bla_TEM_*, *bla_SHV_*, *bla*_CTX__-__M-14_, *bla_CTX__-__M__-__15_*, *bla_OXA_*, *bla_NDM-1_*, *bla_KPC_*, *bla_OXA__-__48_* type, *mcr-1*, *mcr-2*
Jin et al. 2017 [7]	China	16	16	12	Beta-lactams, Quinolones, Aminoglycosides, Sulphonamides, Fosfomycin.	*bla_CTX__-__M-14_*, *bla_CTX__-__M-15_*, *bla_DHA-1_*, *bla_IMP-4_*, *bla_IMP-8_*, *bla_NDM-1_*, *bla_TEM-1_*
John et al. 1983 [32]	United States of America	60	60	60	NR	NR
Keen et al. 2010 [19]	United States of America	2647	695	25	NR	NR
Kim et al. 2020 [12]	South Korea	26	26	26	Beta-lactams, Tetracyclines, Quinolones, Aminoglycosides, Sulphonamides, Polymyxin, Chloramphenicol, Fosfomycin, Nitrofurans.	*aph(3’)-VIa*, *armA*, *aac(6’)-Ib-cr, aadA2*, *aadA1*, *aac(3)-IId*, *strA*, *strB*, *bla_OXA-1_*, *bla_TEM-1A_*, *bla_OXA-9_*, *bla_CTX-M-15_*, *bla_SHV-28_*, *bla_NDM-1_*, *bla_OXA-232_*, *catB3*, *catA1*, *cmlA1*, *mph(E)*, *msr(E)*, *ere(A)*, *qnrB1*, *oqxA*, *oqxB*, *dfrA12*, *dfrA1*, *sul1*, *sul2*, *ARR-3, fosA*, *mgrB*, *phoP*
Kocsis et al. 2014 [17]	Italy	5	3	3	Beta-lactams, Tetracyclines, Quinolones, Aminoglycosides.	*bla_KPC-3_*, *bla_TEM-1_*, *bla_OXA-9_*, *bla_SHV-11_*, *aac(6’)Ib*
Kolpa et al. 2018 [33]	Poland	291	44	10	Beta-lactams, Quinolones, Aminoglycosides.	NR
Kooti et al. 2019 [18]	Iran	150	150	150	Beta-lactams, Quinolones, Aminoglycosides.	*bla_IMP_*, *bla_VIM_*
Lee et al. 2020 [34]	Malaysia	39	36	36	Beta-lactams, Quinolones, Aminoglycosides.	*bla_TEM_*, *bla_SHV_*, *bla_OXA-1_*, *bla_CTX-M-1_*, *bla_CTX-M-9_*
Lima et al. 2014 [35]	Brazil	29	29	21	Beta-lactams, Tetracyclines, Quinolones, Aminoglycosides, Sulphonamides, Chloramphenicol.	NR
Mahmoudi et al. 2017 [36]	Iran	2325	263	200	Beta-lactams, Quinolones, Aminoglycosides, Sulphonamides.	NR
Mansour et al. 2017 [37]	Tunisia	940	220	29	Beta-lactams, Tetracyline, Quinolones, Aminoglycosides, Sulphonamides, Polymyxin, Trimethoprim.	*mgrB*, *bla_OXA-48_*, *bla_OXA-204_*, *bla_CMY-4_*, *bla_NDM-1_*, *bla_CMY-16_*, *bla_CTX-M-15_*
Moges et al. 2019 [38]	Ethiopia	185	97	85	Beta-lactams, Tetracyclines, Quinolones, Aminoglycosides, Sulphonamides, Chloramphenicol.	NR
Nirwati et al. 2019 [39]	Indonesia	962	167	91	Beta-lactams, Quinolones, Aminoglycosides, Sulphonamides.	NR
Okomo et al. 2020 [40]	Gambia	94	6	6	Beta-lactams, Tetracyclines, Quinolones, Aminoglycosides, Sulphonamides.	NR
Oli et al. 2017 [41]	Nigeria	34	5	5	Beta-lactams, Quinolones, Aminoglycosides.	NR
Petro et al. 2014 [42]	Tanzania	172	113	113	Beta-lactams.	NR
Saeed et al. 2010 [10]	Kingdom of Saudi Arabia	710	96	62	Beta-lactams, Quinolones, Aminoglycosides, Sulphonamides, Polymyxin, Chloramphenicol.	NR
Shahi et al. 2019 [43]	Iran	104	104	24	Beta-lactams, Quinolones, Aminoglycosides, Sulphonamides.	*bla_KPC-2_*
Sharahi et al. 2021 [44]	Iran	165	52	5	Beta-lactams, Tetracyclines, Quinolones, Aminoglycosides, Sulphonamides, Fosfomycin.	*bla_TEM_*, *bla_SHV_*, *bla_CTX-M_*, *bla_NDM-1_*, *bla_NDM-6_*
Traub et al. 2000 [45]	Germany	14	14	14	Beta-lactams, Quinolones, Aminoglycosides, Polymyxin, Chloramphenicol, Fosfomycin + Glucose-6-phosphate, Nitrofurantoin, Rifampin	NR
Vaziri et al. 2020 [46]	Iran	126	126	69	Beta-lactams, Quinolones, Aminoglycosides.	*qnrB*, *qnrS*, *aac(6′)-Ib-cr*
Yazdansetad et al. 2019 [47]	Iran	100	100	100	Beta-lactams, Quinolones, Aminoglycosides, Sulphonamides, Nitrofurantoin.	*bla_TEM_*, *bla_CTX-M_*, *bla_SHV_*
Yin et al. 2020 [48]	China	2930	452	134	Beta-lactams, Tetracyclines, Quinolones, Aminoglycosides, Rifamycins.
Zaman et al. 2014 [49]	Kingdom of Saudi Arabia	23	23	23	Beta-lactams, Quinolones, Aminoglycosides, Sulphonamides.	*bla_OXA-48_*, *bla_OXA-D_*, *bla_TEM-1_*, *bla_SHV-1_*, *bla_SHV-11_*, *bla_SHV-12_*, *bla_CTX-M-14_*, *bla_CTX-M-15_*, *aadB*, *dfrA7*
Zeng et al. 2020 [50]	China	37	37	37	Beta-lactams, Quinolones, Aminoglycosides, Sulphonamides.	*bla_KPC-2_*, *bla_SHV_*, *bla_TEM_*, *bla_OXA-1_*, *bla_CTX-M-15_*, *bla_CTX-M-177_*, *bla_CTX-M-3_*, *bla_CTX-M-14_*
Zhong et al. 2012 [51]	China	124	NR	13	Beta-lactams, Quinolones, Aminoglycosides.	NR

NR: Not reported.

**Table 2:** Subgroup analysis of the prevalence of nosocomial MDR *K. pneumoniae* according to countries and regions.

Subgroup	No. of Studies	Prevalence	95% CI	*p*-Value	I^2^ (%)	Q	Heterogeneity Test	
							DF	*p*-Value
Location								
Iraq	2	14.0	2.5–50.5	0.053	94.38	17.793	1	<0.001
Mexico	1	16.7	11.8–23.1	-	-	-	-	-
Iran	10	55.0	27.5–79.8	0.736	98.14	484.982	9	<0.001
United Kingdom	1	95.8	57.5–99.7	-	-	-	-	-
Portugal	1	38.7	23.5–56.5	-	-	-	-	-
**Italy**	**3**	**15.5**	**3.9–45.5**	**0.028**	**94.42**	**35.86**	**2**	**<0.001**
Bangladesh	1	12.0	9.4–15.2	-	-	-	-	-
India	1	6.6	5.7–7.7	-	-	-	-	-
Czech republic	1	72.0	58.1–82.7	-	-	-	-	-
Egypt	1	13.7	8.1–22.2	-	-	-	-	-
**USA**	**4**	**11.6**	**2.5–40.6**	**0.016**	**98.12**	**159.439**	**3**	**<0.001**
Pakistan	1	62.5	55.6–68.9	-	-	-	-	-
China	4	38.6	9.5–79.0	0.612	96.36	82.437	3	<0.001
South Korea	1	98.1	76.4–99.9	-	-	-	-	-
Turkey	1	40.4	33.9–47.2	-	-	-	-	-
Malaysia	1	92.3	78.7–97.5	-	-	-	-	-
Brazil	1	72.4	53.8–85.6	-	-	-	-	-
Tunisia	1	3.1	2.2–4.4	-	-	-	-	-
Brunei	1	91.7	37.8–99.5	-	-	-	-	-
Indonesia	1	9.5	7.8–11.5	-	-	-	-	-
Gambia	1	6.4	2.9–13.5	-	-	-	-	-
Nigeria	1	14.7	6.3–30.8	-	-	-	-	-
Tanzania	1	65.7	58.3–72.4	-	-	-	-	-
Saudi Arabia	2	64.3	0.4–99.9	0.849	94.64	18.640	1	<0.001
Germany	1	96.7	63.4–99.8	-	-	-	-	-
Croatia	1	4.1	1.6–10.5	-	-	-	-	-
Ethiopia	1	45.9	38.9–53.2	-	-	-	-	-
Poland	1	3.4	1.9–6.3	-	-	-	-	-
Region								
Asia	11	39.6	22.1–60.3	0.324	98.35	607.235	10	<0.001
South America	1	72.4	53.8–85.6	-	-	-	-	-
**North America**	**5**	**12.9**	**3.1–** **40.3**	**0.014**	**98.45**	**258.186**	**4**	**<0.001**
Europe	9	31.2	11.5–61.2	0.213	95.84	192.35	8	<0.001
Africa	4	28.7	11.5–55.5	0.114	95.78	71.023	3	<0.001
Middle East	17	35.4	21.2–52.7	0.097	97.79	723.876	16	<0.001

Bold font indicates significant *p*-values. I^1^ represents the heterogeneity in meta-analysis for 47 studies.

## 3. Discussion

This study is the first meta-analysis that estimates the prevalence of antibiotic resistance in nosocomial MDR *K. pneumoniae* worldwide. Here, 47 studies were strictly systematized to be included in this analysis. Based on the included studies, there were 2245 nosocomial MDR *K. pneumoniae* isolates recorded from 28 countries in six regions. A random-effect model was used to analyze the data. As a result, the pooled prevalence of nosocomial MDR *K. pneumoniae* was estimated to be 32.8% (95% CI, 23.6–43.6). A study in Iran showed that the prevalence of nosocomial infections due to *K. pneumoniae* was found at 6.6% (95% CI, 2.1–19.6) [4], which was lower compared to the pooled prevalence of nosocomial MDR *K. pneumoniae* in this study and the prevalence estimate for nosocomial MDR *K. pneumoniae* in Iran, 55.0% (95% CI, 27.5–79.8). While in Ethiopia, the pooled proportional estimates of ESBL-producing *K. pneumoniae* were 61.8% (95% CI, 48.7–73.4) and the pooled proportion estimates of MDR isolates for both *K. pneumoniae* and *E. coli* were 82.7% (95% CI, 72.6–89.6), which was higher when compared with the current study, 45.9% (95% CI, 38.9–53.2). Ethiopia is a resource-limited country where it can be a considerable reason for the high prevalence of *K. pneumoniae* [52].

Other meta-analysis studies under the family of *K. pneumoniae*, *Enterobacteriaceae*, were reported from a few countries and regions. The overall pooled estimate of ESBL-producing *Enterobacteriaceae* was 40% (95% CI, 34.0–47.0) in Pakistan [53] and 42% (95% CI, 34.0–50.0) in East Africa [54], which included five countries. The estimated prevalence of *Enterobacteriaceae* in these two studies was slightly higher than the estimated prevalence of nosocomial MDR *K. pneumoniae* in this analysis. Another study by Mansouri et al. [55] showed a pooled prevalence of ESBL-producing *Enterobacteriaceae* at 25% (95% CI, 18.0–32.0) globally. In addition, the recorded estimated prevalence in Africa, Asia, Europe, South America, and North America was 45% (95% CI, 22.0–67.0), 15% (95% CI, 6.0–24.0), 5% (95% CI, 2.0–8.0), 4% (95% CI, 1.0–11.0), and 3% (95% CI, 1.0–5.0), respectively. This study is likely to share a quite similar result with Cantón et al. [56], where the prevalence of *K. pneumoniae* in the United States of America showed a declining pattern from 7.2% to 4.4%. This result can be proven by Ghashghaee et al. [4], where they estimated that hospital infection rates were between 3.5–12% for the developed countries and between 5.7–19.1% for the developing countries. However, these studies were contradicted with the current study, as the prevalence of nosocomial MDR *K. pneumoniae* in Europe, South America, and North America regions was estimated at 31.2% (95% CI, 11.5–61.2), 72.4% (95% CI, 53.8–85.6), and 12.9% (95% CI, 3.1–40.3), respectively, which was higher than the two studies [55,56]. In this case, the reason that can be proposed was that the transmission of nosocomial MDR *K*. *pneumoniae* strains was higher in these regions. Eastern and South-Western Europe, together with the Mediterranean countries, are endemic to MDR *K. pneumoniae*, due to the ESBL strain. In these countries, the resistance rates were more than 50–60% for the third generation of cephalosporins, fluoroquinolones, and aminoglycosides antibiotic classes [57].

Based on the included studies, the information on antibiotic resistance profiles and the responsible genes encoded for antibiotic resistance were tabulated in Table 1 and Figure 2. Based on Figure 2A, 43 (91.49%), 41 (87.23%), 40 (85.11%), 24 (51.06%), 18 (38.30%), 16 (34.04%), and 7 (14.89%) of the included studies showed consistent resistance to beta-lactams, quinolones, aminoglycosides, sulphonamides, other classes of antibiotics, tetracyclines, and polymyxins, respectively. All of the MDR *K. pneumoniae* isolates from nosocomial infections were completely resistant to aminopenicillins, penicillins with inhibitors of β-lactamases (except piperacillin/tazobactam), cephalosporins, quinolones, tigecycline, and tobramycin [58]. While a study by Hou et al. [59] stated the high prevalence in resistance to aminoglycosides, macrolides, quinolones, and beta-lactams MDR *K. pneumoniae* isolates. Furthermore, a majority of the included studies described the resistant genes encoded for β-lactamases enzymes as contributing to the high resistance to the class of beta-lactams.

Since the treatment for patients infected with MDR *K. pneumoniae* is quite challenging and costly, this high prevalence estimate is alarming enough. There were some efficient treatments used in treating the patients with MDR *K. pneumoniae* in hospitals, such as colistin [60], fosfomycin [58], and double carbapenem therapy [61]. Furthermore, risk factors that contribute to nosocomial infections, such as poor hygienic conditions in healthcare settings, immunosuppression in patients, extended days in intensive care units (ICU), prolonged consumption of antibiotics, inappropriate use of injection techniques and invasive devices (catheters), a lack of knowledge of basic infection control measures, and inadequacy of control policies, must be addressed and managed in order to control this emergence [62].

There were several limitations in this study. First, this analysis does not cover all of the countries in order to understand the complete overview of prevalence in nosocomial MDR *K. pneumoniae,* which is due to the lack of resources for some countries. In addition, the analysis on antibiotic resistance profiles could not be conducted due to the varied antibiotics that were used, although they were the same classes of antibiotics in the included studies. Therefore, the highest prevalence of antibiotic classes that contribute to the nosocomial MDR *K*. *pneumoniae* could not be identified in this study.

## 4. Materials and Methods

### 4.1. Selection Criteria

All of the articles related to the isolates of MDR *K. pneumoniae* from clinical samples of patients with nosocomial infections were considered for inclusion. The articles with isolates of MDR *K. pneumoniae* from patients with community-acquired infection, MDR *K. pneumoniae* from the environment and animals, non-MDR *K. pneumoniae*, other *Klebsiella* spp. isolated from patients, as well as unrecoverable full texts and unrelated results were excluded from this study.

### 4.2. Literature Search

A combination of specific keywords in the title or abstract, such as “*Klebsiella pneumoniae*” AND (“antibiotic resistance” OR “antibiotic susceptibility”) were used during the search in four electronic databases: PubMed, ScienceDirect, Google Scholar, and Scopus.

The analysis was conducted on those studies that had enough information to answer the objective. In the initial screening, the abstracts were screened by three authors (NAMA, NMH, NFMZ) based on the inclusion and exclusion criteria. The articles that fulfilled the selection criteria were included and proceeded for full-text screening. Disagreement was resolved by discussion and was further verified by two authors (NYY, SA).

This study was registered in the International Prospective Register of Systematic Reviews (PROSPERO), with a registered ID of CRD42021262133.

### 4.3. Data Extraction and Quality Assessment

Data extraction was conducted based on the objective. In this study, the following data were extracted from the included studies: URL, authors, publication year, study design, study period, country, number, gender and age of patients, types of diseases, area of infection, types of samples, total number of isolates, total number of *K. pneumoniae* isolates, total number of MDR *K. pneumoniae* isolates, total number of antibiotics tested, percentage of resistance and susceptibility to antibiotic classes (Beta-lactams, tetracyclines, quinolones, aminoglycosides, sulphonamides, polymyxins, and others), percentage of MDR *K. pneumoniae*, type of treatment to MDR *K. pneumoniae,* and genes encoded for antibiotic resistance.

The quality of eligible studies was assessed with the Joanna Brigs Institute (JBI) Quality Assessment Tool for prevalence studies. The quality scores (proportion) were computed for all of the articles. The risk of bias was considered low when more than 70% of the answers were “yes”, moderate when 50–69% of the answers were “yes”, and high when less than 50% of the answers were “yes”. The articles with a low risk of bias only were included in the studies, while the articles that showed a high and moderate risk of bias were excluded from the review.

### 4.4. Data Synthesis and Data Analysis

Data analysis was performed using OpenMeta Analyst. The pooled prevalence of nosocomial MDR *K. pneumoniae* was measured and the subgroup analysis was performed according to the location and geographical region. A random-effect model using the DerSimonian-Laird method of meta-analysis was used to create the pooled estimates of the reported nosocomial MDR *K. pneumoniae* cases. The potential publication bias was examined by creating a funnel plot with the comprehensive meta-analysis and the asymmetry of the plot was further assessed using Egger’s regression test. The heterogeneity of the study-level estimates was evaluated using Cochran’s Q test and I^2^ statistic as outlined: 0 to 40% might not be important; 30 to 60% may represent moderate heterogeneity; 50 to 90% may represent substantial heterogeneity; and 75 to 100% would be considerable heterogeneity [63]. A sensitivity test using the leave-one-out analysis was conducted. For all of the tests, a *p*-value of < 0.05 was considered to be statistically significant.

## 5. Conclusions

In conclusion, based on the 28 countries in six regions from the included studies, this meta-analysis showed that the pooled prevalence of nosocomial MDR *K. pneumoniae* was estimated at 32.8% (95% CI, 23.6–43.6), which was considerably moderate, but the emergence of this pathogen was accelerated. Based on the 47 studies, all of the studies with an antibiotic resistance profile showed high resistance to the beta-lactams class (*n* = 43), followed by quinolones (*n* = 41), and aminoglycosides class (*n* = 40). Most of the studies recorded for resistance genes showed the resistance genes that were encoded for beta-lactams and they likely contributed to the antibiotic resistance in the class of beta-lactams. Immediately, the proper protocol needs to be emphasized in the healthcare setting to ensure that the emergence and spread of the nosocomial MDR *K*. *pneumoniae* can be prevented.

## Data Availability

The datasets used and/or analyzed during the current study are included in the manuscript.

## Appendix A

**Table A1:** Quality of the included studies by the JBI critical appraisal checklist for studies reporting prevalence data.

No	Author ID	Checklist ^1^									Overall Score
		1	2	3	4	5	6	7	8	9	
1.	Abdul et al. 2020	Yes	No	Yes	Yes	Yes	Yes	Yes	Yes	Yes	8
2.	Abdul Momin et al. 2017	Yes	No	Yes	Yes	Yes	Yes	Yes	No	Yes	7
3.	Alcántar-Curiel et al. 2018	Yes	No	Yes	Yes	No	Yes	Yes	Yes	Yes	7
4.	Aljanaby and Alhasani 2016	Yes	No	Yes	Yes	No	Yes	Yes	Yes	Yes	7
5.	Amani et al. 2020	Yes	No	Yes	Yes	Yes	Yes	Yes	Yes	Yes	8
6.	Anes et al. 2017	Yes	No	Yes	Yes	No	Yes	Yes	Yes	Yes	7
7.	Ashayeri-Panah et al. 2014	Yes	No	Yes	Yes	No	Yes	Yes	Yes	Yes	7
8.	Badamchi et al. 2018	Yes	Yes	Yes	Yes	No	Yes	Yes	Yes	Yes	8
9.	Bandic-Pavlovic et al. 2020	Yes	Yes	Yes	Yes	No	Yes	Yes	Yes	Yes	8
10	Bidell et al. 2017	Yes	Yes	Yes	Yes	No	Yes	Yes	Yes	Yes	8
11.	Caneiras et al. 2019	Yes	No	Yes	Yes	No	Yes	Yes	Yes	Yes	7
12.	Chakraborty et al. 2016	Yes	No	Yes	Yes	No	Yes	Yes	Yes	Yes	7
13.	Das and Debnath 2015	Yes	No	Yes	Yes	Yes	Yes	Yes	Yes	Yes	8
14.	Dolejska et al. 2012	Yes	Yes	Yes	No	Yes	Yes	Yes	Yes	Yes	8
15.	Durdu et al. 2019	Yes	Yes	Yes	Yes	Yes	Yes	Yes	Yes	Yes	9
16.	Eghbalpoor et al. 2019	Yes	Yes	Yes	Yes	Yes	Yes	Yes	Yes	Yes	9
17.	Eid et al. 2020	Yes	Yes	Yes	No	Yes	Yes	Yes	Yes	Yes	8
18.	Folgori et al. 2014	Yes	Yes	Yes	Yes	Yes	No	Yes	No	Yes	7
19.	Giufre et al. 2018	Yes	No	Yes	Yes	Yes	Yes	Yes	Yes	Yes	8
20.	Glasser et al. 2010	Yes	No	Yes	Yes	No	Yes	Yes	Yes	Yes	7
21.	Imtiaz et al. 2021	No	Yes	Yes	No	Yes	Yes	Yes	Yes	Yes	7
22.	Jin et al. 2017	Yes	No	Yes	Yes	Yes	Yes	Yes	No	Yes	7
23.	John et al. 1983	Yes	No	Yes	Yes	Yes	Yes	Yes	No	Yes	7
24.	Keen et al. 2010	Yes	No	Yes	Yes	Yes	Yes	Yes	Yes	Yes	8
25.	Kim et al. 2020	Yes	No	Yes	Yes	Yes	Yes	Yes	Yes	Yes	8
26.	Kocsis et al. 2014	Yes	Yes	No	No	Yes	Yes	Yes	Yes	Yes	7
27.	Kolpa et al. 2018	Yes	No	Yes	Yes	Yes	Yes	Yes	No	Yes	7
28.	Kooti et al. 2019	Yes	Yes	Yes	No	Yes	Yes	Yes	Yes	Yes	8
29.	Lee et al. 2020	Yes	Yes	Yes	No	Yes	Yes	Yes	No	Yes	7
30.	Lima et al. 2014	Yes	Yes	Yes	No	Yes	Yes	Yes	No	Yes	7
31.	Mahmoudi et al. 2017	Yes	No	Yes	Yes	Yes	Yes	Yes	Yes	Yes	8
32.	Mansour et al. 2017	Yes	No	Yes	Yes	Yes	Yes	Yes	No	Yes	7
33.	Moges et al. 2019	Yes	No	Yes	Yes	Yes	Yes	Yes	No	Yes	7
34.	Nirwati et al. 2019	Yes	No	Yes	Yes	No	Yes	Yes	Yes	Yes	7
35.	Okomo et al. 2020	Yes	No	Yes	Yes	No	Yes	Yes	Yes	Yes	7
36.	Oli et al. 2017	Yes	No	Yes	Yes	No	Yes	Yes	Yes	Yes	7
37.	Petro et al. 2014	Yes	No	Yes	Yes	Yes	Yes	Yes	Yes	Yes	8
38.	Saeed et al. 2010	Yes	Yes	Yes	No	Yes	Yes	Yes	Yes	Yes	8
39.	Shahi et al. 2019	Yes	Yes	Yes	No	Yes	Yes	Yes	Yes	Yes	8
40.	Sharahi et al. 2021	Yes	Yes	Yes	Yes	Yes	Yes	Yes	Yes	Yes	9
41.	Traub et al. 2000	Yes	Yes	Yes	No	Yes	Yes	Yes	Yes	Yes	8
42.	Vaziri et al. 2020	Yes	Yes	Yes	Yes	Yes	Yes	Yes	Yes	Yes	9
43.	Yazdansetad et al. 2019	Yes	Yes	Yes	Yes	Yes	Yes	Yes	Yes	Yes	9
44.	Yin et al. 2020	Yes	Yes	Yes	Yes	Yes	Yes	Yes	Yes	Yes	9
45.	Zaman et al. 2014	Yes	Yes	Yes	No	Yes	Yes	Yes	Yes	Yes	8
46.	Zeng et al. 2020	Yes	Yes	Yes	Yes	Yes	Yes	Yes	No	Yes	8
47.	Zhong et al. 2012	Yes	Yes	Yes	Yes	Yes	Yes	Yes	Yes	Yes	9

^1^ The checklist questions to determine the risk of bias for the included studies: 1. Was the sample frame appropriate to address the target population? 2. Were the study participants sampled in an appropriate way? 3. Was the sample size adequate? 4. Were the study subjects and the setting described in detail? 5. Was a sample size justification, power description or variance and effect estimates provided? 6. Were valid methods used for the identification of the condition? 7. Was the condition measured in a standard, reliable way for all participants? 8. Was there an appropriate statistical analysis? 9. Was the response rate adequate, and if not, was the low response rate managed appropriately?

**Table A2:** The list of antibiotic classes under Beta-lactams with resistance to MDR *K. pneumoniae* in 47 studies.

Author ID	Antibiotic Resistance to the Beta-Lactams Class
Abdul et al. 2020	Beta-lactams; Penicillins, Penicillins/Beta-lactamase inhibitor, Cephalosporins, Carbapenems, Monobactams.
Abdul Momin et al. 2017	Beta-lactams; Cephalosporins, Carbapenems.
Alcántar-Curiel et al. 2018	Beta-lactams; Penicillins, Cephalosporins.
Aljanaby and Alhasani 2016	Beta-lactams; Penicillins, Cephalosporins, Carbapenems.
Amani et al. 2020	Beta-lactams; Penicillins, Cephalosporins, Carbapenems.
Anes et al. 2017	Beta-lactams; Penicillins, Penicillins/Beta-lactamase inhibitor, Cephalosporins, Carbapenems, Monobactams.
Ashayeri-Panah et al. 2014	Beta-lactams; Penicillins, Penicillins/Beta-lactamase inhibitor, Cephalosporins, Monobactams.
Badamchi et al. 2018	Beta-lactams; Penicillins, Penicillins/Beta-lactamase inhibitor, Cephalosporins, Carbapenems.
Bandic-Pavlovic et al. 2020	Beta-lactams; Penicillins, Penicillins/Beta-lactamase inhibitor, Cephalosporins, Carbapenems.
Bidell et al. 2017	Beta-lactams; Penicillins/Beta-lactamase inhibitor, Cephalosporins, Carbapenems.
Caneiras et al. 2019	Beta-lactams; Penicillins/Beta-lactamase inhibitor, Cephalosporins.
Chakraborty et al. 2016	Beta-lactams; Penicillins, Cephalosporins.
Das and Debnath 2015	Beta-lactams; NR
Dolejska et al. 2012	Beta-lactams; Carbapenems.
Durdu et al. 2019	Beta-lactams; Penicillins/Beta-lactamase inhibitor, Cephalosporins, Cephalosporins/Beta-lactamase inhibitor, Carbapenems.
Eghbalpoor et al. 2019	Beta-lactams; Penicillins, Penicillins/Beta-lactamase inhibitor, Cephalosporins, Carbapenems.
Eid et al. 2020	Beta-lactams; Penicillins/Beta-lactamase inhibitor, Cephalosporins, Carbapenems.
Folgori et al. 2014	Beta-lactams; NR
Giufre et al. 2018	Beta-lactams; Penicillins, Cephalosporins.
Glasser et al. 2010	Beta-lactams; Penicillins/Beta-lactamase inhibitor, Cephalosporins.
Imtiaz et al. 2021	Beta-lactams; Penicillins, Cephalosporins, Carbapenems, Monobactams.
Jin et al. 2017	Beta-lactams; Penicillins/Beta-lactamase inhibitor, Cephalosporins, Carbapenems, Monobactams.
John et al. 1983	Beta-lactams; NR
Keen et al. 2010	Beta-lactams; NR
Kim et al. 2020	Beta-lactams; Penicillins, Penicillins/Beta-lactamase inhibitor, Cephalosporins, Carbapenems, Monobactams.
Kocsis et al. 2014	Beta-lactams; Cephalosporins, Carbapenems, Monobactams.
Kolpa et al. 2018	Beta-lactams; Penicillins, Penicillins/Beta-lactamase inhibitor, Cephalosporins, Cephalosporins/Beta-lactamase inhibitor, Carbapenems.
Kooti et al. 2019	Beta-lactams; Cephalosporins, Carbapenems, Monobactams.
Lee et al. 2020	Beta-lactams; Penicillins, Cephalosporins.
Lima et al. 2014	Beta-lactams; Penicillins, Penicillins/Beta-lactamase inhibitor, Cephalosporins, Carbapenems, Monobactams.
Mahmoudi et al. 2017	Beta-lactams; Penicillins, Penicillins/Beta-lactamase inhibitor, Cephalosporins, Carbapenems.
Mansour et al. 2017	Beta-lactams; Penicillins, Penicillins/Beta-lactamase inhibitor, Cephalosporins, Carbapenems, Monobactams.
Moges et al. 2019	Beta-lactams; Penicillins, Cephalosporins.
Nirwati et al. 2019	Beta-lactams; Penicillins, Penicillins/Beta-lactamase inhibitor, Cephalosporins, Carbapenems.
Okomo et al. 2020	Beta-lactams; Penicillins, Cephalosporins.
Oli et al. 2017	Beta-lactams; Penicillins, Cephalosporins.
Petro et al. 2014	Beta-lactams; Penicillins/Beta-lactamase inhibitor.
Saeed et al. 2010	Beta-lactams; Penicillins, Penicillins/Beta-lactamase inhibitor, Cephalosporins, Carbapenems, Monobactams.
Shahi et al. 2019	Beta-lactams; Cephalosporins, Carbapenems.
Sharahi et al. 2021	Beta-lactams; Penicillins, Penicillins/Beta-lactamase inhibitor, Cephalosporins, Carbapenems, Monobactams.
Traub et al. 2000	Beta-lactams; Penicillins, Penicillins/Beta-lactamase inhibitor, Cephalosporins, Carbapenems, Monobactams.
Vaziri et al. 2020	Beta-lactams; Cephalosporins, Monobactams.
Yazdansetad et al. 2019	Beta-lactams; Cephalosporins, Carbapenems.
Yin et al. 2020	Beta-lactams; Penicillins/Beta-lactamase inhibitor, Cephalosporins, Cephalosporins/Beta-lactamase inhibitor, Carbapenems.
Zaman et al. 2014	Beta-lactams; Penicillins/Beta-lactamase inhibitor, Cephalosporins, Carbapenems.
Zeng et al. 2020	Beta-lactams; Penicillins/Beta-lactamase inhibitor, Cephalosporins, Carbapenems
Zhong et al. 2012	Beta-lactams; Penicillins, Penicillins/Beta-lactamase inhibitor, Cephalosporins.

NR is not recorded.
